# No association between germline allele-specific expression of *TGFBR1* and colorectal cancer risk in Caucasian and Ashkenazi populations

**DOI:** 10.1038/sj.bjc.6606079

**Published:** 2011-02-15

**Authors:** N Seguí, K N Stevens, E Guinó, L S Rozek, V R Moreno, G Capellá, S B Gruber, L Valle

**Affiliations:** 1 Translational Research Laboratory, Catalan Institute of Oncology, IDIBELL, Hospitalet de Llobregat, Av. Gran Vía 199-203, Barcelona 08908, Spain; 2 Departments of Internal Medicine, Epidemiology, and Human Genetics, Division of Molecular Medicine and Genetics, University of Michigan, 1524 BSRB, 109 Zina Pitcher, Ann Arbor, MI 48109, USA; 3 Bioinformatics and Biostatistics Unit, Catalan Institute of Oncology, IDIBELL, Hospitalet de Llobregat, Barcelona 08908, Spain; 4 Environmental Health Sciences, School of Public Health, University of Michigan Medical School, Ann Arbor, MI 48109, USA; 5 Department of Clinical Sciences, Faculty of Medicine, University of Barcelona, Barcelona, Spain

**Keywords:** allele-specific expression, allelic imbalance, *TGFBR1*, colorectal cancer predisposition, pyrosequencing, cancer-predisposing gene

## Abstract

**Background:**

Germline allele-specific expression (ASE) of the *TGFBR1* gene has been reported as a strong risk factor for colorectal cancer (CRC) with an odds ratio close to 9. Considering the potential implications of the finding, we undertook the task of validating the initial results in this study.

**Methods:**

Allele-specific expression was measured using the highly quantitative and robust technique of pyrosequencing. Individuals from two different populations were studied, one Caucasian-dominated and the other of Ashkenazi Jewish descent, with different sources of non-tumoral genetic material in each.

**Results:**

Our results showed no statistically significant differences in the degree of ASE between CRC patients and controls, considering ASE as either a quantitative or a binary trait. Using defined cutoff values to categorise ASE, 1.0% of blood lymphocytes from informative Israeli cases (total *n*=96) were ASE positive (median 1.00; range 0.76–1.31) and 2.2% of informative matched controls (total *n*=90) were ASE positive (median 1.00; range 0.76–1.87). Likewise, normal mucosae from Spanish patients (median 1.03; range: 0.68–1.43; *n*=75) did not show significant differences in the degree of ASE when compared with the Israeli patients or controls.

**Conclusions:**

Taken together, these results suggest that ASE of *TGFBR1* does not confer an increased risk of CRC.

Colorectal cancer (CRC) is among the four most common cancers in industrialised countries, and is one of the leading causes of cancer-related deaths. Although familial clustering of CRC occurs in 20–30% of all cases, the known highly penetrant autosomal-dominant and -recessive forms of the disease account for less than 5% of all CRC cases (de la Chapelle, 2004; Lynch *et al*, 2009). Although additional low-penetrance alleles have been proposed in the last few years, the underlying genetic risk factors for CRC predisposition remain largely unknown (Hemminki *et al*, 2009).

On the basis of previous evidence that pointed towards the importance of downstream signalling elements of the transforming growth factor *β* (TGF-*β*) pathway in CRC (Wood *et al*, 2007), and the known linkage peak for familial CRC in 9q22–31 where *TGFBR1* is located (Wiesner *et al*, 2003; Kemp *et al*, 2006; Skoglund *et al*, 2006), we undertook the task of studying the role of *TGFBR1* in CRC predisposition. Although risk-conferring germline genetic variants in this gene had not been identified, we reported that germline allele-specific expression (ASE) of *TGFBR1*, measured with the SNaPshot technique, occurred in ∼20% of informative CRC patients and ∼3% of informative controls, thus conferring a substantially increased risk of CRC (odds ratio 8.7, 95% confidence interval (CI): 2.6–29.1) (Valle *et al*, 2008). This differential allele-specific expression was suggested to be dominantly inherited and to alter the downstream SMAD-mediated TGF-*β* signalling (Valle *et al*, 2008). A subsequent report showed that *APC*^*Min*/+^;*Tgfbr1*^+/−^ mice developed twice as many intestinal tumours and colonic carcinomas as *APC*^*Min*/+^;*Tgfbr1*^+/+^, supporting the role of *TGFBR1* gene haploinsufficiency in CRC development (Zeng *et al*, 2009). Also, TGFBR1^*^6A, a common variant in exon 1 of the gene, has been weakly associated with CRC (Pasche *et al*, 2004; Skoglund *et al*, 2007).

As allele-specific expression of *TGFBR1* has the potential to be used in the clinical evaluation of CRC risk, the aim of this study was to further investigate the extent of ASE of *TGFBR1* in CRC using the robust and specific pyrosequencing technique for ASE determination. In addition, we studied two different populations with different biological sources of non-tumour genetic material to evaluate ASE frequency in a variety of populations.

## Materials and methods

### Patients and controls

Uncultured blood lymphocytes from a total of 426 Ashkenazi Jewish CRC patients and 433 Ashkenazi Jewish controls were obtained from a collection of Israeli CRC patients and matched controls. This series corresponds to a population-based case–control study (Molecular Epidemiology of Colorectal Cancer; MECC) of incident CRC, including histopathologically confirmed cases of all incident CRC diagnosed in northern Israel beginning 31 March 1998 (Poynter *et al*, 2005). Informed consent was obtained from all of the subjects who participated in the study. All 426 CRC patients showed tumour microsatellite stability and did not carry germline mutations in known cancer-predisposing genes.

A total of 178 normal mucosae from Spanish CRC patients (6% showed tumour microsatellite instability) were obtained from a hospital-based case–control study (Bellvitge Colorectal Cancer Study; BCCS). Cases were consecutive patients with a new diagnosis of colorectal adenocarcinoma attending a University Hospital in Barcelona. Details about the study population, interviews and collection of biological samples were published elsewhere (Landi *et al*, 2003).

### Nucleic acid extraction and cDNA synthesis

Genomic DNA from purified blood lymphocytes and frozen normal colon mucosa was extracted using standard phenol–chloroform procedures. For total RNA extraction, the different tissue sources were processed with TRIzol reagent (Invitrogen, Carlsbad, CA, USA). In all cases, nucleic acid concentrations and purities were analyzed with the NanoDrop spectrophotometer, and the level of degradation of the RNA (RIN number) was checked by using the RNA Nano assay on the Agilent 2100 Bioanalyzer system (Agilent Technologies, Santa Clara, CA, USA) when necessary.

Total RNA was treated with DNAse (DNAfree, Ambion, Austin, TX, USA) before cDNA synthesis (Transcriptor First Strand cDNA Synthesis Kit, Roche Diagnostics GmbH, Mannheim, Germany).

### Genotyping of transcribed SNPs

Current techniques for ASE determination require heterozygous markers in the transcribed regions of the gene to discriminate between its two alleles. The transcribed markers we used were four SNPs located in the 3′-UTR region: rs334349, rs420549, rs7850895 and rs1590. Owing to the fact that rs334349 and rs1590 are in strong linkage disequilibrium, only the latter was genotyped to determine the informativeness of both. Three commercially available TaqMan SNP genotyping assays were used to genotype rs420549 (C_662618_1), rs7850895 (C_29248567_20) and rs1590 (C_2945143_10) (Applied Biosystems Inc., Foster City, CA, USA). Reactions were performed following the instructions provided by the manufacturer.

### ASE determination by pyrosequencing

PCR and pyrosequencing reactions for rs334349, rs1590 and rs420549 were performed as described previously (Guda *et al*, 2009). For rs7850895, PCR and sequencing primers were designed using the PSQ Assay Design software provided by the manufacturer: PCR-fw-5′-TCATGCCATATGTAGTTGCTGTAG-3′ biotinylated PCR-rv-5′-ACACCCCTAAGCATGTGGAGA-3′ and SEQ-5′-CCTAGTGCAAGTTACAATAT-3′. After PCR, DNA and cDNA amplification products were sequenced on a PyroMark MD pyrosequencing instrument (Qiagen, Chatsworth, CA, USA).

The proportions of individual alleles for each SNP were obtained from the PyroMark MD software calculations. The medians and standard deviations (s.d.) of the triplicates for both DNA and cDNA were calculated for each SNP. To obtain an ASE value, the ratio of the common *vs* the rare allele in the cDNA was normalised to the respective ratio in the DNA: cDNA (median common allele/median rare allele)/DNA (median common allele/median rare allele). The final ASE value was calculated as the median of the ASE values obtained for the SNPs studied in each sample.

Before the complete analysis of all informative samples, we tested the robustness and reproducibility of pyrosequencing compared with SNaPshot, the technique used in the original report (Valle *et al*, 2008), by randomly choosing 10 informative samples and measuring ASE using both techniques. SNaPshot was carried out as described previously (Valle *et al*, 2008) and ASE value calculations were performed as described above for both techniques. Pyrosequencing yielded lower variability in ASE among different SNP markers and it was able to obtain valuable results in situations when SNaPshot was not able to assess ASE (Supplementary Table 1). Guda *et al* (2009) previously reported additional information showing that ASE results obtained by pyrosequencing can be reproduced by SNaPshot, supporting the ability of pyrosequencing to detect allelic imbalances.

### Statistical analyses

Pairwise comparisons between cases and controls were performed using the Wilcoxon's rank-sum test and Bonferroni correction was applied to account for the two comparisons performed: MECC cases *vs* MECC controls and MECC cases *vs* BCCS cases.

To dichotomise the ASE variable, cutoff points were established based on the ASE values obtained in cancer-free controls (median±(2 × s.d.)). When ASE was considered as a binary variable, comparisons of proportions between cases and controls were performed using a likelihood ratio test derived from logistic regression adjusting for population source.

## Results

Among a total of 426 Ashkenazi Jewish CRC patients from the Israeli MECC study, 115 (27%) were informative for at least one SNP tested: 88 (20.7%) for rs334349 and rs1590, 65 (15.3%) for rs420549 and 18 (4.2%) for rs7850895. Of a total of 433 Ashkenazi Jewish MECC controls, 112 (25.9%) were informative: 81 (18.7%) for rs334349 and rs1590, 59 (13.6%) for rs420549 and 19 (4.4%) for rs7850895. Of 178 normal mucosae from a Spanish collection of Caucasian CRC patients (BCCS), 88 (49.4%) were heterozygous for at least one SNP genotyped: 64 (36%) for rs334349 and rs1590, 64 (36%) for rs420549 and 18 (10%) for rs7850895. Three of the 88 had no RNA available.

**Figure 1:**
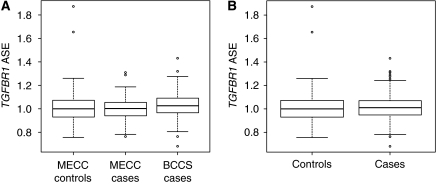
*TGFBR1* ASE distributions in cases and controls. (**A**) *TGFBR1* ASE in MECC controls (*n*=90), MECC CRC patients (*n*=96) and BCCS CRC patients (*n*=75). (**B**) *TGFBR1* ASE in CRC patients (*n*=171) and controls (*n*=90). The boxes represent the inter-quartile range of distributions (25–75th percentile); the horizontal lines within the boxes represent the medians; and the vertical lines represent the 5 and 95th percentiles.

**Figure 2:**
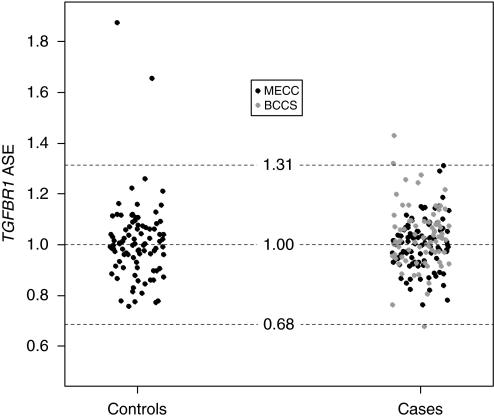
*TGFBR1* ASE distribution in 171 CRC patients (96 MECC (black dots) and 75 BCCS (grey dots) CRC patients) and 90 controls. The median and cutoff points, defined as the median±2 × s.d. of controls, used to categorise ASE are indicated as discontinuous lines.

ASE values were obtained for 96 (83.5%) informative MECC CRC patients, 90 (80.4%) informative MECC controls and 75 (85.2%) informative BCCS CRC patients. The ASE values obtained for cases and controls are shown in Figure 1. For the MECC series alone, values range from 0.76 to 1.31 (median: 1.00) in cases, and from 0.76 to 1.87 (median: 1.00) in controls (Figure 1A). When ASE was considered as a continuous variable, no differences were detected between cases and controls (median difference −0.002; 95% CI: −0.027 to 0.032; *P*=0.86). Although observed data suggest that ASE is a quantitative trait, ASE was transformed into a binary trait (ASE *vs* non-ASE) to facilitate the interpretation of the results. For this purpose, cutoff points were defined based on the results obtained in controls (median±(2 × s.d)=1.00±(2 × 0.157)). Under that criterion, 1.0% (1 out of 96) of informative CRC patients and 2.2% (2 out of 90) of informative controls showed ASE of *TGFBR1* (*P*=0.52) (Figure 2).

No differences in ASE levels were identified between MECC CRC patients (median 1.00; range 0.76–1.31) and BCCS patients (median 1.03; range 0.68–1.43) (median difference 0.026; 95% CI: −0.001 to 0.059; *P*=0.06). Consistent with this analysis, no differences were identified between MECC and BCCS CRC patients when ASE was treated as a binary variable (*P*=0.20), suggesting that ethnic origin (Ashkenazi Jewish and Caucasian) and the source of biological material assessed (uncultured blood lymphocytes and normal colon mucosae) do not have a major influence on *TGFBR1* ASE assessment. This allowed us to combine the two groups of CRC patients and compare them with the available group of controls (Figures 1B and 2). Combining all data from MECC and BCCS subjects, no differences were detected between CRC patients and controls when considering ASE as either a quantitative (median difference −0.010; 95% CI: −0.037 to 0.017; *P*=0.48) or a binary variable (*P*=0.52, adjusted by population).

The RNA quality of subjects with ASE in both the BCCS and MECC series was checked to confirm that they had been classified as such owing to the presence of real allelic imbalances and not owing to technical artefacts caused by poor RNA quality. In all samples where the source RNA was available, the RIN value was above 6. To ensure that our results were not affected by poor performance of the PCR/pyrosequencing reaction owing to low RNA quality, a more stringent analysis was carried out, including only those samples whose s.d. among pyrosequencing triplicates was below 0.20. Similar to the results using the entire sample, no differences were detected between CRC patients and controls (Supplementary Figure 1).

## Discussion

The presence of allelic imbalances is well known to be widespread throughout the transcriptome and has been associated with cancer risk in some instances (Yan *et al*, 2002; Raval *et al*, 2007; Chen *et al*, 2008). In a previous report, we suggested that ASE of *TGFBR1* confers a substantially increased risk of CRC (odds ratio 8.7), potentially placing ASE of *TGFBR1* among the major contributors to the genetic predisposition to both familial and sporadic CRC (Valle *et al*, 2008). The main significance of those findings pertains to early detection and prevention of CRC, therefore requiring validation in larger series and different populations for future implementation in clinical practice. Here, using a more robust technique for ASE determination, studying Ashkenazi Jewish and Caucasian populations, and using different sources of non-tumoral genetic material, we identified no differences in the degree or frequency of ASE of *TGFBR1* between CRC patients and controls, discarding its role in CRC predisposition. Our results are supported by the study by Guda *et al* (2009), in which it was concluded that ASE of *TGFBR1* is unlikely to be the major driver of linkage in some colon neoplasia families to the 9q22.2–31.2 region, in which *TGFBR1* is located, and that ASE is not associated with sporadic CRC (*n*=44). Recently, Carvajal-Carmona *et al* (2010) reported no evidence of genetic variation at *TGFBR1* as a predisposing factor for CRC and found no increased level of *TGFBR1* ASE in 24 familial CRC patients compared with 45 informative controls. In fact, ASE turned out to be more prevalent among controls than among cases.

Very recently, two additional studies on ASE of *TGFBR1* were published. In the first study, ASE of TGFBR1, assessed by SNaPshot, was found in approximately 10% of CRC patients (15% of informative patients), agreeing with our initial report; however, no controls were included for comparison (Pasche *et al*, 2010). In the second study, where ASE was measured by pyrosequencing, 109 informative cases and 125 informative controls were studied. No differences were identified when ASE was considered as a binary variable; however, when treated with a continuous variable, ASE was significantly higher in cases than in controls. However, the differences identified between CRC patients and controls were very subtle and definitely not useful for cancer risk assessment (Tomsic *et al*, 2010). Table 1 shows the main characteristics and results of previous studies focused on the role of ASE of *TGFBR1* in CRC risk.

Several features differentiate the original and present studies, and important consequences might have derived from these differences. The use of different assays for ASE determination and the exclusion of cases that showed high variability among replicates (exclusion of samples with low-quality source RNA shown in Supplementary Material) have likely increased the robustness of our study. Tomsic *et al* (2010) also found that the SNaPshot technology used for ASE determination gave inconsistent results, as evidenced by considerably larger standard deviations compared with pyrosequencing, and that high RNA quality is essential for reproducibility of ASE.

Some SNP markers and sources of nucleic acids used were also different. The rs7871490 SNP, located in the 3′-UTR of *TGFBR1*, was used as a marker in the original study (Valle *et al*, 2008), but not in the present one. We previously found that the marker was very useful because it allowed us to significantly increase the number of informative individuals from 40 to 55–60%. This SNP is located in a region of repetitive sequence, 5′-GGGGGTTTTTTTTTTGTTTTTTTTTT[G/T]TTGTTGTTGTTTTTGGGCCATTTCT-3′, which might have affected the correct performance of SNaPshot owing to the design and molecular basis of the technique. When excluding all individuals from the original study whose ASE value was only based on the results obtained from the analysis of rs7871490 (16 out of 29 CRC patients and 3 out of 3 controls with ASE values >1.5), the proportion of ASE in informative CRC patients drops from 21% (29 out of 138) to 13% (13 out of 97) and in informative controls from 3% (3 out of 105) to 0% (0 out of 76). Likely because of the repetitive sequences in the flanking region of rs7871490, we were not able to design a pyrosequencing assay, which precluded a direct comparison between SNaPshot and pyrosequencing. A subset of individuals within this group belonged to the so-called ‘group 2’, which was characterised by a particular haplotype significantly over-represented among ASE CRC patients (Valle *et al*, 2008). To increase the number of informative individuals, the rs420549 and rs7850895 allelic markers were included in this study. This resulted in an increase of 22 out of 75 (29%) informative BCCS CRC patients (three of which showed ASE), 22 out of 96 (23%) informative MECC CRC patients (one showed ASE) and 28 out of 90 (31%) informative MECC controls (two showed ASE). In short, all ASE individuals were informative for only either rs420549 or rs7850895. This observation, together with what has been discussed above about rs7871490, suggests that ASE might be more common among individuals who carry minor alleles for specific *TGFBR1* SNPs, and therefore might be more or less frequent depending on the panel of SNP markers used to define informative individuals. Nevertheless, our results point to a similar frequency of ASE among cases and controls.

The possibility of ASE being tissue specific has been suggested previously (Cowles *et al*, 2002; Wilkins *et al*, 2007). This was one concern that arose in the paper from Guda *et al* (2009), in which the sources of nucleic acids for ASE determination were EBV-transformed cultured lymphocytes and normal mucosae from CRC patients, in contrast to the total blood used in our initial study (Valle *et al*, 2008). Carvajal-Carmona *et al* (2010) also employed lymphoblastoid cell lines. It is still unknown whether EBV transformation and/or cell culture alter the allelic expression of genes. Here, we obtained uncultured blood lymphocytes from the MECC series, which may well correlate with the total blood used in our previous study (Valle *et al*, 2008) or by Tomsic *et al* (2010), and normal mucosae from BCCS patients, which can be compared with the sporadic cases reported in the series from Guda *et al* (2009). These results suggest that different (uncultured) biological sources of genetic material for the determination of *TGFBR1* ASE can be used without distinction.

In the original report, a mostly Caucasian population from Central Ohio was evaluated, whereas Ashkenazi Israeli and Caucasian Spanish populations were studied here. The fact that ASE first seemed to be heavily dependent on allele frequencies left open the possibility of inter-ethnic variation. The degree of SNP informativity was different between the two populations (20% MECC *vs* 36% BCCS for rs334349 and rs1590; 14% MECC *vs* 36% BCCS for rs420549; and 4% MECC *vs* 10% BCCS for rs7850895); however, no differences were detected in the level of ASE between the two populations. Unfortunately, we did not have access to all types of samples (normal mucosae and lymphocytes) from the same individuals or populations; therefore, there remains a certain degree of uncertainty about tissue and ethnic variability.

In conclusion, the improved determination of ASE of *TGFBR1* achieved by pyrosequencing revealed no differences between CRC cases and controls, in both Caucasian and Ashkenazi populations. The sample size in ASE studies is highly relevant owing to their dependence on marker informativity and to the difficulties associated with collection of high-quality germline RNA. Finally, the use of different sources of non-tumour nucleic acids for ASE determination adds consistency to our results. However, the lack of informativity for transcribed SNPs in a substantial proportion of individuals complicates the task to assess the extent of germline ASE of *TGFBR1* in CRC. New technological advances that allow the measurement of allelic imbalances in a more precise and informative manner will be of substantial importance to provide a definitive answer to the real extent of ASE at *TGFBR1* in CRC patients.

## Figures and Tables

**Table 1:** Summary of the characteristics and results obtained in different studies on ASE of *TGFBR1* and CRC risk

						**ASE: Cases *vs* controls^a^**	
**Study**	**Population**	**Nucleic acid source**	**Methodology**	**Allelic markers**	**Informative cases; controls**	**Quantitative**	**Binary (ASE *vs* non-ASE)**
Valle *et al* (2008)	C ∼90% O ∼10%	Peripheral blood	SNaPshot	rs334348 rs7871490 rs334349 rs1590	138; 105	*P*=0.12 Wilcoxon test *P*=0.02 Permutation test	Cutoff: <0.67, ⩾1.5 (ROC analysis) ASE: 29/138; 3/105 *P*=7.66 × 10^−5^
Guda *et al* (2009)	C ∼89% AA ∼9% O ∼2%	Lymphoblastoid cell line (EBV) Normal colon mucosa	Pyrosequencing	rs868 rs334348 rs334349 rs420549 rs1590	Familial: 46; 17 Sporadic: 44; 0	N/A	Cutoff: <0.67, ⩾1.5 (Valle *et al*, 2008) Familial ASE: 2/46; 0/17 Sporadic ASE: 0/44
Carvajal-Carmona *et al* (2010)	C ∼100%	Lymphoblastoid cell line (EBV)	Genescan SNaPshot	*6A/9A rs1590	Familial: 24; 45	*P*=0.09 Wilcoxon test^b^ *P*=0.13 *t*-test^b^	Cutoff: <0.67, ⩾1.5 (Valle *et al*, 2008) ASE: 7/24; 12/45 *P*=0.83
Pasche *et al* (2010)	C ∼83% AA ∼12% O ∼5%	Lymphoblastoid cell line (EBV)	SNaPshot	rs334348 rs7871490 rs334349 rs1590	74; 0	N/A	Cutoff: <0.67, ⩾1.5 (Valle *et al*, 2008) ASE: 11/74
Tomsic *et al* (2010)	C ∼92% AA ∼8%	Peripheral blood	Pyrosequencing	rs868 rs334348 rs334349 rs420549 rs1590	109; 125^c^	*P*=0.009;0.006 Wilcoxon test *P*=0.081;0.077 Permutation test * P*=0.003;0.007 Permutation (median)	Cutoff: <0.67, ⩾1.5 (Valle *et al*, 2008) ASE: 2/109; 2/125 Cutoff: <0.9, ⩾1.1 (ROC analysis) ASE: 51/109; 39/125 *P*=0.06
This study	Ash 100% C 100%	Uncultured lymphocytes (Ash) Normal colon mucosa (C)	Pyrosequencing	rs334349 rs7850895 rs420549 rs1590	Ash: 96; 90 C: 75; 0	Ash: *P*=0.86 Wilcoxon (median) Ash+C: *P*=0.48 Wilcoxon (median)	Cutoff: <0.67, ⩾1.5 (Valle *et al*, 2008) Ash ASE: 0/96; 2/90 Ash+C ASE: 0/171; 2/90 Cutoff: <0.68, ⩾1.31 (median±2 × s.d.) Ash ASE: 1/96; 2/90 *P*=0.52 Ash+C ASE: 4/171; 2/90 *P*=0.52

Abbreviations: AA=African-American; ASE=allele-specific expression; Ash=Ashkenazi Jewish; C=Caucasian; CRC=colorectal cancer; EBV=Epstein–Barr virus; NA=not available; O=Others; ROC=receiver operating characteristic.

a ASE measured as (A_cDNA_/B_cDNA_)/(A_gDNA_/B_gDNA_), A being the common allele and B the rare allele.

b ASE trend was more pronounced in the controls than in the cases.

c Forty-nine cases were the same as in Valle *et al* (2008).

## References

[bib1] Carvajal-Carmona LG, Churchman M, Bonilla C, Walther A, Lefevre JH, Kerr D, Dunlop M, Houlston R, Bodmer WF, Tomlinson I. Comprehensive assessment of variation at the transforming growth factor beta type 1 receptor locus and colorectal cancer predisposition. Proc Natl Acad Sci USA (2010); 107: 7858–7862 20368424 10.1073/pnas.1002816107 PMC2867909

[bib2] Chen X, Weaver J, Bove BA, Vanderveer LA, Weil SC, Miron A, Daly MB, Godwin AK (2008) Allelic imbalance in BRCA1 and BRCA2 gene expression is associated with an increased breast cancer risk. Hum Mol Genet 17: 1336–1348 18204050 10.1093/hmg/ddn022

[bib3] Cowles CR, Hirschhorn JN, Altshuler D, Lander ES (2002) Detection of regulatory variation in mouse genes. Nat Genet 32: 432–437 12410233 10.1038/ng992

[bib4] de la Chapelle A (2004) Genetic predisposition to colorectal cancer. Nat Rev Cancer 4: 769–780 15510158 10.1038/nrc1453

[bib5] Guda K, Natale L, Lutterbaugh J, Wiesner GL, Lewis S, Tanner SM, Tomsic J, Valle L, de la Chapelle A, Elston RC, Willis J, Markowitz SD (2009) Infrequent detection of germline allele-specific expression of TGFBR1 in lymphoblasts and tissues of colon cancer patients. Cancer Res 69: 4959–4961 19509225 10.1158/0008-5472.CAN-09-0225 PMC2739986

[bib6] Hemminki K, Forsti A, Lorenzo Bermejo J (2009) Surveying the genomic landscape of colorectal cancer. Am J Gastroenterol 104: 789–790 19262529 10.1038/ajg.2008.100

[bib7] Kemp ZE, Carvajal-Carmona LG, Barclay E, Gorman M, Martin L, Wood W, Rowan A, Donohue C, Spain S, Jaeger E, Evans DG, Maher ER, Bishop T, Thomas H, Houlston R, Tomlinson I (2006) Evidence of linkage to chromosome 9q22.33 in colorectal cancer kindreds from the United Kingdom. Cancer Res 66: 5003–5006 16707420 10.1158/0008-5472.CAN-05-4074

[bib8] Landi S, Moreno V, Gioia-Patricola L, Guino E, Navarro M, de Oca J, Capella G, Canzian F (2003) Association of common polymorphisms in inflammatory genes interleukin (IL)6, IL8, tumor necrosis factor alpha, NFKB1, and peroxisome proliferator-activated receptor gamma with colorectal cancer. Cancer Res 63: 3560–3566 12839942

[bib9] Lynch HT, Lynch PM, Lanspa SJ, Snyder CL, Lynch JF, Boland CR (2009) Review of the Lynch syndrome: history, molecular genetics, screening, differential diagnosis, and medicolegal ramifications. Clin Genet 76: 1–18 10.1111/j.1399-0004.2009.01230.x PMC2846640 19659756

[bib10] Pasche B, Kaklamani V, Hou N, Young T, Rademaker A, Peterlongo P, Ellis N, Offit K, Caldes T, Reiss M, Zheng T (2004) TGFBR1^*^6A and cancer: a meta-analysis of 12 case–control studies. J Clin Oncol 22: 756–758 14966109 10.1200/JCO.2004.99.271

[bib11] Pasche B, Wisinski KB, Sadim M, Kaklamani V, Pennison MJ, Zeng Q, Bellam N, Zimmerman J, Yi N, Zhang K, Baron J, Stram DO, Hayes MG (2010) Constitutively decreased TGFBR1 allelic expression is a common finding in colorectal cancer and is associated with three TGFBR1 SNPs. J Exp Clin Cancer Res 29: 57 20500843 10.1186/1756-9966-29-57 PMC2890549

[bib12] Poynter JN, Gruber SB, Higgins PD, Almog R, Bonner JD, Rennert HS, Low M, Greenson JK, Rennert G (2005) Statins and the risk of colorectal cancer. N Engl J Med 352: 2184–2192 15917383 10.1056/NEJMoa043792

[bib13] Raval A, Tanner SM, Byrd JC, Angerman EB, Perko JD, Chen SS, Hackanson B, Grever MR, Lucas DM, Matkovic JJ, Lin TS, Kipps TJ, Murray F, Weisenburger D, Sanger W, Lynch J, Watson P, Jansen M, Yoshinaga Y, Rosenquist R, de Jong PJ, Coggill P, Beck S, Lynch H, de la Chapelle A, Plass C (2007) Downregulation of death-associated protein kinase 1 (DAPK1) in chronic lymphocytic leukemia. Cell 129: 879–890 17540169 10.1016/j.cell.2007.03.043 PMC4647864

[bib14] Skoglund J, Djureinovic T, Zhou XL, Vandrovcova J, Renkonen E, Iselius L, Bisgaard ML, Peltomaki P, Lindblom A (2006) Linkage analysis in a large Swedish family supports the presence of a susceptibility locus for adenoma and colorectal cancer on chromosome 9q22.32–31.1. J Med Genet 43: e7 16467217 10.1136/jmg.2005.033928 PMC2564647

[bib15] Skoglund J, Song B, Dalen J, Dedorson S, Edler D, Hjern F, Holm J, Lenander C, Lindforss U, Lundqvist N, Olivecrona H, Olsson L, Pahlman L, Rutegard J, Smedh K, Tornqvist A, Houlston RS, Lindblom A (2007) Lack of an association between the TGFBR1^*^6A variant and colorectal cancer risk. Clin Cancer Res 13: 3748–3752 17575241 10.1158/1078-0432.CCR-06-2865

[bib16] Tomsic J, Guda K, Liyanarachchi S, Hampel H, Natale L, Markowitz SD, Tanner SM, de la Chapelle A. Allele-specific expression of TGFBR1 in colon cancer patients. Carcinogenesis (2010); 31(10): 1800–1804 20705955 10.1093/carcin/bgq165 PMC2950937

[bib17] Valle L, Serena-Acedo T, Liyanarachchi S, Hampel H, Comeras I, Li Z, Zeng Q, Zhang HT, Pennison MJ, Sadim M, Pasche B, Tanner SM, de la Chapelle A (2008) Germline allele-specific expression of TGFBR1 confers an increased risk of colorectal cancer. Science 321: 1361–1365 18703712 10.1126/science.1159397 PMC2672914

[bib18] Wiesner GL, Daley D, Lewis S, Ticknor C, Platzer P, Lutterbaugh J, MacMillen M, Baliner B, Willis J, Elston RC, Markowitz SD (2003) A subset of familial colorectal neoplasia kindreds linked to chromosome 9q22.2–31.2. Proc Natl Acad Sci USA 100: 12961–12965 14566058 10.1073/pnas.2132286100 PMC240727

[bib19] Wilkins JM, Southam L, Price AJ, Mustafa Z, Carr A, Loughlin J (2007) Extreme context specificity in differential allelic expression. Hum Mol Genet 16: 537–546 17220169 10.1093/hmg/ddl488

[bib20] Wood LD, Parsons DW, Jones S, Lin J, Sjoblom T, Leary RJ, Shen D, Boca SM, Barber T, Ptak J, Silliman N, Szabo S, Dezso Z, Ustyanksky V, Nikolskaya T, Nikolsky Y, Karchin R, Wilson PA, Kaminker JS, Zhang Z, Croshaw R, Willis J, Dawson D, Shipitsin M, Willson JK, Sukumar S, Polyak K, Park BH, Pethiyagoda CL, Pant PV, Ballinger DG, Sparks AB, Hartigan J, Smith DR, Suh E, Papadopoulos N, Buckhaults P, Markowitz SD, Parmigiani G, Kinzler KW, Velculescu VE, Vogelstein B (2007) The genomic landscapes of human breast and colorectal cancers. Science 318: 1108–1113 17932254 10.1126/science.1145720

[bib21] Yan H, Dobbie Z, Gruber SB, Markowitz S, Romans K, Giardiello FM, Kinzler KW, Vogelstein B (2002) Small changes in expression affect predisposition to tumorigenesis. Nat Genet 30: 25–26 11743581 10.1038/ng799

[bib22] Zeng Q, Phukan S, Xu Y, Sadim M, Rosman DS, Pennison M, Liao J, Yang GY, Huang CC, Valle L, Di Cristofano A, de la Chapelle A, Pasche B (2009) Tgfbr1 haploinsufficiency is a potent modifier of colorectal cancer development. Cancer Res 69: 678–686 19147584 10.1158/0008-5472.CAN-08-3980 PMC2668823

