# Supplementary material for: No association between germline allele-specific expression of TGFBR1 and colorectal cancer risk in Caucasian and Ashkenazi populations
Source: Br J Cancer. 2011 Feb 15;104(4):735–40. doi: 10.1038/sj.bjc.6606079 (PMC3049588; doi:10.1038/sj.bjc.6606079)
Supplement: Supplementary Material [file 6606079x1.doc]

SUPPLEMENTARY MATERIAL

Supplementary Table 1. Results of the pilot study with samples from 10 informative CRC patients. ASE results obtained with the four 3’UTR SNPs used, an overall ASE value calculated as the median of the ASE values for the informative SNPs, and a standard deviation (s.d) value that indicates the variability among different SNPs, are shown for both SNaPshot and pyrosequencing assays.

| **Samples** | BCCS1 | BCCS2 | BCCS3 | BCCS4 | BCCS5 | BCCS6 | BCCS7 | BCCS8 | MECC1 | MECC2 |
| --- | --- | --- | --- | --- | --- | --- | --- | --- | --- | --- |
| **SNaPshot** | | | | | | | | | | |
| **rs334349** | 1.37 | NV | 0.92 |  | 0.79 |  | 0.91 | 1.06 |  | 0.95 |
| **rs1590** | 0.94 | NV | 0.61 |  | 0.54 |  | 0.74 | 0.72 |  | 0.65 |
| **rs420549** | 1.06 | NV | 1.05 | 1.06 | 1.29 |  |  | 1.35 | 1.31 | 1.05 |
| **rs7850895** |  |  |  |  |  | NV |  |  |  |  |
| **ASE (median)** | 1.06 |  | 0.92 | 1.06 | 0.79 |  | 0.83 | 1.06 | 1.31 | 0.95 |
| **s.d.** | 0.22 |  | 0.23 | - | 0.38 |  | 0.12 | 0.32 | - | 0.25 |
| **Pyrosequencing** | | | | | | | | | | |
| **rs334349** | 1.04 | 0.81 | 1.03 |  | 1.08 |  | 1.08 | 1.05 |  | 0.98 |
| **rs1590** | 1.01 | NV | 1.09 |  | 0.90 |  | 0.98 | 0.85 |  | 0.67 |
| **rs420549** | 1.00 | NV | 1.03 | 1.08 | 1.04 |  |  | 1.13 | 0.85 | 1.24 |
| **rs7850895** |  |  |  |  |  | 0.97 |  |  |  |  |
| **ASE (median)** | 1.01 | 0.81 | 1.03 | 1.08 | 1.04 | 0.97 | 1.03 | 1.05 | 0.85 | 0.98 |
| **s.d.** | 0.02 | - | 0.03 |  | 0.09 | - | 0.07 | 0.14 | - | 0.29 |

**Controls**

**Cases**

1.24

1.00

0.76

BCCS

MECC

Supplementary Figure 1. *TGFBR1* ASE distribution in 156 CRC patients (70 MECC (black dots) and 86 BCCS (grey dots) CRC patients) and 75 controls. The median and cutoff points, defined as the median +/- 2*s.d. of controls, used to categorize ASE are indicated as discontinuous lines. Those samples with s.d. among pyroseqeuncing triplicates above 0.20 were excluded from the analysis. No differences were detected between MECC cases and MECC controls (median difference -0.003; 95%CI -0.032 to 0.025; P=0.81); between MECC cases and BCCS cases (median difference 0.016; 95%CI -0.015 to 0.047; P=0.32); and between all cases and controls (median difference 0.010; 95% CI -0.038 to 0.015; P=0.41). Also, no statistically significant differences were detected among all groups of cases and controls when ASE was categorized as a binary variable.
